# Uterine Dehiscence in the Early Third Trimester: A Report of Two Cases

**DOI:** 10.7759/cureus.40911

**Published:** 2023-06-24

**Authors:** Kristina Nalbandyan, Tina Bui, Kristina Roloff, Guillermo J Valenzuela

**Affiliations:** 1 Obstetrics and Gynecology, Arrowhead Regional Medical Center, Colton, USA

**Keywords:** ultrasound detection of uterine dehiscence, cesarean delivery, uterine thickness, uterine dehiscence, uterine rupture

## Abstract

As the incidence of cesarean deliveries increases, so do its accompanying complications. Although the incidence of uterine dehiscence in the late second trimester to the early third trimester is rare, it may be a potentially catastrophic complication if uterine rupture occurs. Here, we present two cases of uterine dehiscence at 28 and 29 weeks, which were diagnosed on prenatal ultrasound and confirmed intraoperatively at the time of cesarean delivery. We recommend consideration of earlier screening for preoperative detection of uterine dehiscence to help prevent maternal and neonatal morbidity and mortality.

## Introduction

The overall rate of cesarean deliveries is on the rise in the USA, with a national rate of 32.1% reported in 2021 [1]. Many of these are primary cesarean deliveries, and therefore the total number of cesarean deliveries performed annually is projected to increase. This will lead to an increase in complications associated with multiple uterine surgeries, including uterine dehiscence and uterine rupture. In uterine dehiscence, the visceral peritoneum is intact, and the fetus remains in the uterine cavity. In uterine rupture, the uterine peritoneum is completely disrupted and the fetus is outside of the uterine cavity. Uterine dehiscence is most often diagnosed at the time of repeat cesarean section in the late third trimester; however, dehiscence may be present but undetected much earlier putting the patient at risk for uterine rupture. Uterine rupture in the second trimester is rare and thought to occur in around one in 5,000 cases [2]. Prenatal ultrasound sensitivity has been shown to be 67.5% for uterine dehiscence [2]. Although there is no general consensus regarding their management, we recommend consideration of earlier screening for preoperative detection of uterine dehiscence in women with high-order multiple prior cesarean deliveries to help prevent maternal and neonatal morbidity and mortality.

## Case presentation

Case 1

**Figure 1:**
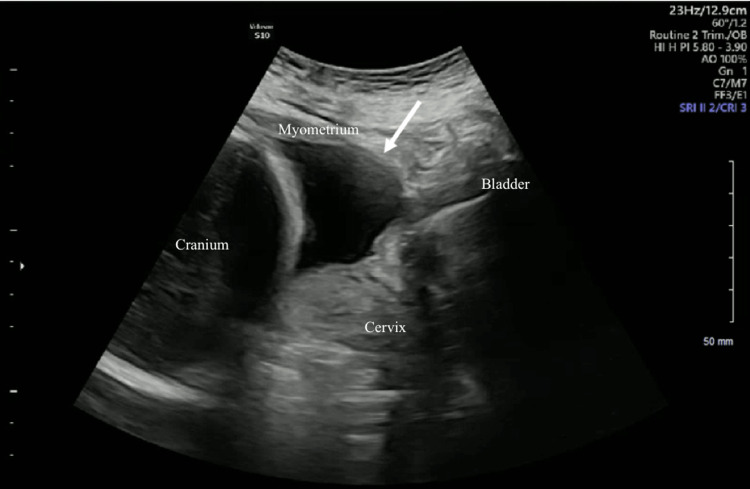
Transabdominal ultrasound of the lower uterine segment. Transabdominal ultrasound of the lower uterine segment showing the myometrium anteriorly abruptly thinning with only a serosal layer remaining with protrusion of the amniotic sac through this defect in the sagittal view.

A 39-year-old G11 P3-1-6-4 woman with three previous cesarean deliveries presented at 28^4/7^ weeks for preterm contractions and was admitted to our Labor and Delivery Department for observation. The patient’s vital signs were normal on initial presentation, and pelvic examination showed a closed and long cervix. A category 1 fetal heart rate tracing was noted, and no uterine contractions were detected on tocometry. Of note, the patient had a history of uterine dehiscence in her second cesarean delivery but not at the time of her third cesarean delivery. The second cesarean delivery was performed six years after her first delivery as a scheduled term repeat delivery. The patient was counseled on the risks of short-interval pregnancy; however, she became pregnant eight months after her second delivery and six months after her third delivery. On admission, a maternal-fetal medicine (MFM) ultrasound was performed, which suggested lower uterine segment dehiscence. The myometrium anteriorly was noted to be abruptly thin with minimal visible tissue and bulging of the lower uterine segment anteriorly toward the bladder (Figure 1).

**Figure 2:**
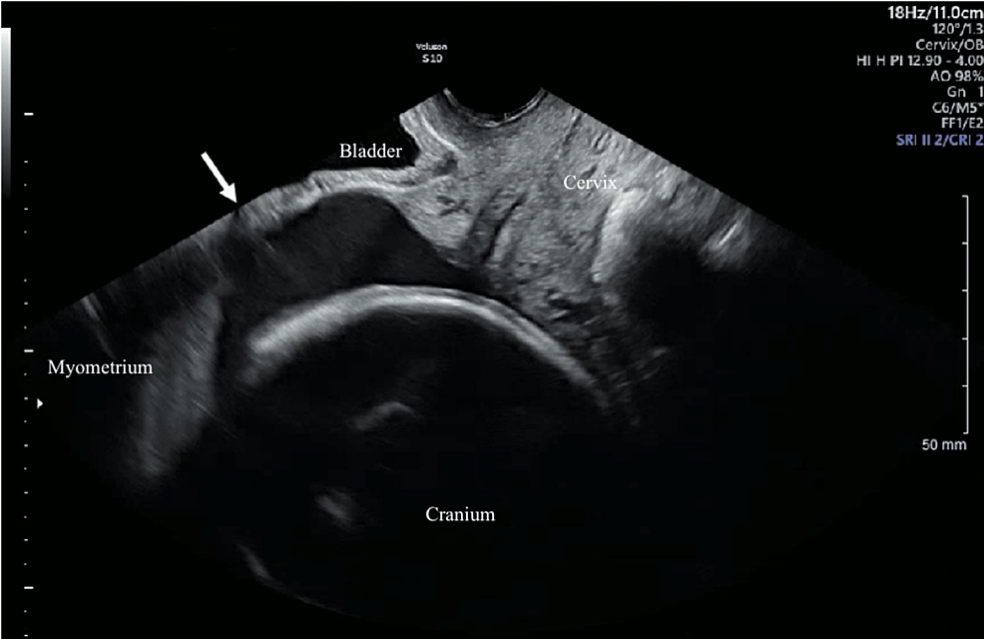
Transvaginal ultrasound of the lower uterine segment. Transvaginal sonographic image of the lower uterine segment re-demonstrating the presence of uterine dehiscence of 3.7 cm in the sagittal view

This defect measured approximately 4 cm (Figure 2). 

As the fetal heart rate tracing remained reassuring, the patient was given betamethasone 12 mg intramuscularly every 12 hours for two doses for fetal lung maturity and was started on tocolysis with indomethacin 25 mg every 6 hours for 48 hours. Indomethacin was recommended by the MFM team over alternative tocolytics given the gestational age. Four days later, the patient complained of uterine contractions, and the decision was made to proceed with repeat cesarean delivery at 29^2/7^ weeks due to potential risk of uterine rupture. Magnesium sulfate 4 g IV bolus was given prior for neuroprotection. Intraoperatively, the patient was found to have a 5- to 6-cm uterine scar dehiscence. A female fetus was delivered through a high transverse incision, with Apgar scores of 6 at 1 minute, 7 at 5 minutes, and 9 at 10 minutes of life, with a birthweight of 1,350 g. The baby spent 40 days in the neonatal intensive care unit (NICU) and was discharged home, with no documented complications until present. The patient had a normal operative quantitative blood loss of 737 mL but was transfused two units of packed red blood cells following delivery because the pre-operative hemoglobin was 7.7 g/dL.

Case 2

**Figure 3:**
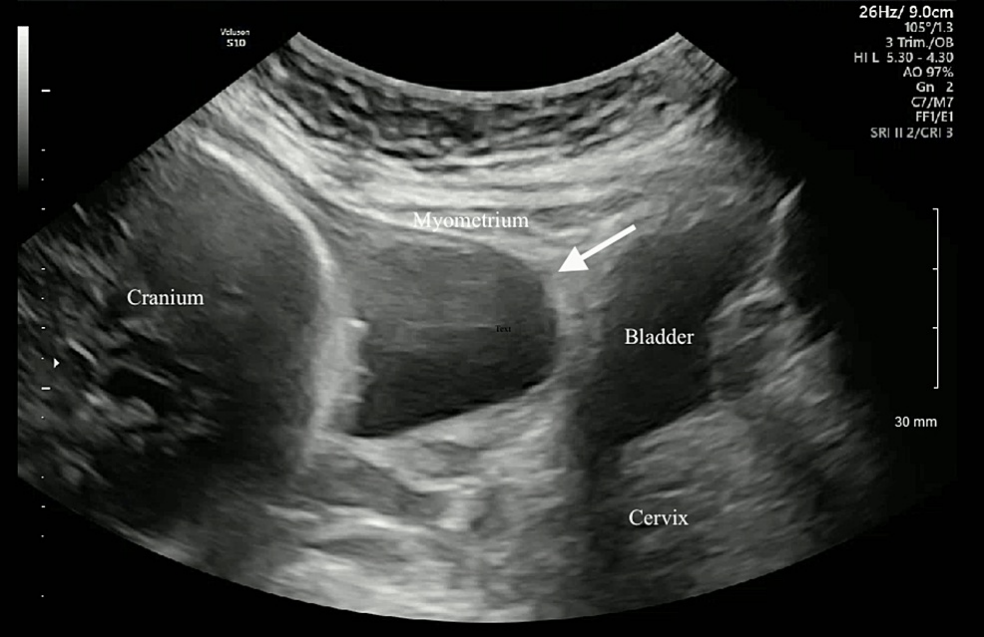
Transabdominal sonographic image of the lower uterine segment showing the presence of a thin myometrium that tapers off and herniation of the amniotic sac toward the bladder in the sagittal view.

A 36-year-old G5 P4-0-0-4 woman with three previous cesarean deliveries was admitted for observation to our Labor and Delivery Department at 29^2/7^ weeks after ultrasound performed by an MFM specialist suggested a uterine dehiscence. Of note, her prior delivery was only 14 months ago. Our MFM practice routinely evaluates patients with high-order uterine surgeries for signs of placenta accreta spectrum at the 18- to 20-week ultrasound and again in the early third trimester. The patient was asymptomatic. The ultrasound showed marked myometrial thinning in the lower uterine segment, with a window that measured approximately 1.1 mm thick and approximately 3.7 cm long. A pelvic MRI was performed for confirmation, which corroborated the ultrasound findings of a focal anterior lower uterine segment bulge with wall thinning (Figure 3). The patient was given betamethasone 12 mg intramuscularly every 12 hours for two doses for fetal lung maturity and monitored for symptoms. A repeat ultrasound two weeks later revealed no visible myometrial tissue present and bulging of the amniotic membranes anteriorly toward the bladder.

**Figure 4:**
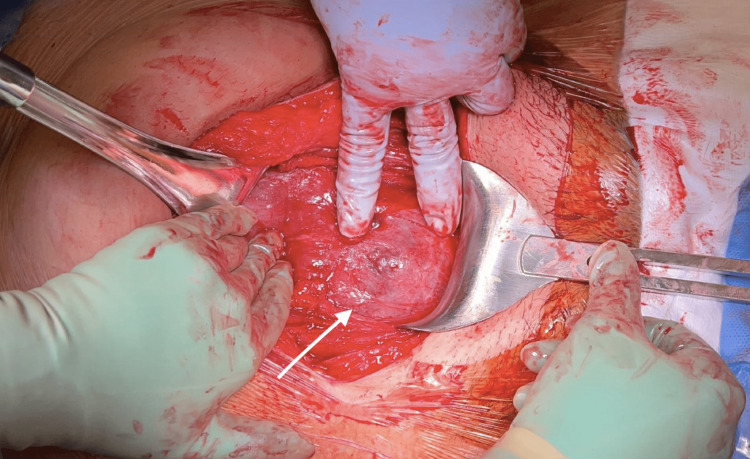
Operative view of uterine dehiscence. Operative view of uterine dehiscence, measuring 5x3 cm, after entering the peritoneum with bladder adhesions.

Due to concern for uterine rupture, the decision was made to proceed with repeat cesarean delivery at 31 weeks. Magnesium sulfate 4 g IV bolus was started at this time for neuroprotection. Intraoperatively, she was noted to have a 5x3 cm uterine dehiscence (Figure 4).

A male fetus was delivered through a high transverse incision with Apgar scores of 8 at 1 minute of life and 9 at 10 minutes of life, with a birthweight of 1,690 g. The baby spent 52 days in the NICU and was discharged home with no complications until present.

## Discussion

An undiagnosed uterine dehiscence can lead to uterine rupture and profound complications for both the mother and the fetus, such as hemorrhage requiring blood transfusion, hysterectomy, and maternal or fetal death [3]. Pre-operative detection of uterine dehiscence prior to uterine rupture may help prevent morbidity and mortality. There are several risk factors for uterine scar dehiscence, including prior uterine surgery, history of prior uterine dehiscence or rupture, short-interval pregnancy, advanced maternal age, and anemia. Prior uterine surgery (especially multiple prior cesarean deliveries) is the leading risk factor for a uterine rupture or dehiscence [4,5]. Patients with a history of uterine dehiscence are also cautioned to have another pregnancy as the rates of recurrence may be as high as 33% [6]. The risk of uterine rupture was higher in patients undergoing a trial of labor with an interpregnancy interval of fewer than six months [7]. In all seven cases of uterine rupture reported by Ramphal and Moodley, there was a degree of mild anemia [5]. Lastly, a retrospective study performed by Shipp et al. concluded that women aged 30 years or older have a greater risk of uterine rupture than their younger counterparts [8].

The optimal gestational age and frequency for screening for scar dehiscence is not known. A literature review suggests that most institutions measure lower uterine segment thickness in the late third trimester, mostly between 34 and 36 weeks’ gestation, in patients at risk for uterine rupture. However, there are several case reports of uterine rupture in the late second to early third trimester, which suggests that screening earlier may help detection rates [9,10]. While risks of uterine rupture and dehiscence are directly related to lower uterine segment thickness as measured on antenatal ultrasound, there is no consensus on lower uterine segment thickness thresholds, and screening for lower uterine segment thickness has not been adopted widely into clinical practice. Rozenberg et al. proposed that a lower uterine segment thickness of >3.5 mm confers protection, and values less than that have variable rates of uterine rupture, including 10% with values between 2.6 and 3.5 mm and 16% with values between 1.6 and 2.5 mm [11]. Cui and Wu also found a 16.8% rate of uterine rupture in myometrial thickness of <1 mm [12]. In addition, myometrial thickness is not static and changes through pregnancy, most rapidly decreasing between the second and third trimesters; thus, close interval follow-up and serial ultrasounds are indicated [13].

## Conclusions

There is no consensus on optimal delivery timing once a uterine scar dehiscence has been diagnosed on antenatal ultrasound, especially in women in the late second or early third trimester of pregnancy. Factors that contribute to the decision-making process of preterm delivery include gestational age and the risks of developmental sequelae of prematurity, fetal demise, uterine rupture, and preterm labor. In the handful of case reports in the literature of individuals undergoing conservative management of uterine dehiscence, two cases underwent emergent delivery at 31 and 33 weeks for fetal distress, one case required emergent delivery at 28 weeks after preterm premature rupture of membranes with fetal parts protruding through the site of rupture, and one case reported excess bleeding. Thus, planned cesarean delivery is predicted to provide significantly better maternal and neonatal outcomes as compared to expectant management with emergency cesarean delivery. Further research is needed to determine if implementing universal lower uterine segment ultrasound screening for scar dehiscence in patients with significant risk for uterine rupture in the early third trimester will help prevent the catastrophic events that may follow uterine rupture, but this case report demonstrates that prenatal antenatal ultrasound can predict uterine dehiscence, especially in patients who are symptomatic.
